# Koroška 8000: digit responses to cold stress following himalayan expedition to broadpeak, Pakistan (8051 m)

**DOI:** 10.1186/2046-7648-4-S1-A43

**Published:** 2015-09-14

**Authors:** Jurij Gorjanc, Shawnda A Morrison, Adam C McDonnell, Jan Babič, Igor B Mekjavic

**Affiliations:** 1 Department of Surgery, Hospital of the Brothers of St. John of God, Spitalgasse 26, A-9300 Sankt Veit an der Glan, Austria; 2 Science and Research Centre, University of Primorska, Garibaldijeva 1, Koper, Slovenia; 3 Department of Automation, Biocybernetics and Robotics, Jožef Stefan Institute, Jamova 39, SI-1000 Ljubljana, Slovenia; 4 Jozef Stefan International Postgraduate School, Jamova Cesta 39, 1000 Ljubljana, Slovenia

## Introduction

We investigated the effects chronic hypobaric hypoxia exposure would have on alpinists' physiological adaptations, including: aerobic fitness, body composition, haematological variables and digit perfusion responses to cold stress, performed before and immediately after a 35 day high altitude climbing expedition.

## Methods

Seven elite Slovenian alpinists completed a battery of physiological tests, including a cold stress test protocol previously used to determine changes in digit temperatures [1-3]. Briefly, alpinists immersed their hand or foot (random order) into a circulated, warm water bath (35°C) for 5 min to standardise skin temperature, and then in a cold water bath (8°C) for 30 min. Individual digit temperatures (thermocouples) were measured continuously for each min during the protocol, and for an additional 10 min of passive recovery in air.

## Results

5/7 alpinists successfully summited Broadpeak (8051 m elevation). Of those alpinists, 4/5 demonstrated higher cold-induced vasodilation (CIVD) wave amplitudes in mean finger temperatures, or higher recovery temperatures, (or both), post-expedition. In the feet, 1/5 had higher wave amplitudes, 1/5 had higher passive recovery temperatures, whereas 2/5 had lower mean toe temperatures during cold exposure, and one had no discernible alterations post-expedition. One alpinist declined participation in the cold stress testing in this expedition because he had previously completed an identical protocol and reported extreme discomfort in his digits during the cold water immersion phase of testing. Area under the curve calculations for the hands found 5/5 alpinists had higher values post-expedition, whilst in the toes, 3/5 had higher values compared to pre-expedition.

## Discussion

Previous results have demonstrated a significant enhancement of the CIVD response in both fingers and toes of alpinists returning from high altitude expeditions 1 and following 15-months of military training in the cold, 4 whilst others have reported variable differences following repeated cold exposure, depending on altitude 5. Vasodilatation and vasoconstriction responses are non-generalisable between hands and feet 3. It is not clear whether uniform peripheral cold adaptation *per se *occurs in both hands and feet following combined exposure to high altitude and cold in this particular population.

## Conclusion

Alpinists presented vastly different digit responses to cold stress after exposure to hypobaric hypoxia.
